# Molecular Evidence for a Thymus-Independent Partial T Cell Development in a FOXN1^−/−^ Athymic Human Fetus

**DOI:** 10.1371/journal.pone.0081786

**Published:** 2013-12-09

**Authors:** Anna Fusco, Luigi Panico, Marisa Gorrese, Gabriella Bianchino, Maria V. Barone, Vitina Grieco, Laura Vitiello, Roberta D’Assante, Rosa Romano, Loredana Palamaro, Giulia Scalia, Luigi Del Vecchio, Claudio Pignata

**Affiliations:** 1 Department of Translational Medical Sciences, Pediatric Section, “Federico II” University, Naples, Italy; 2 Unit of Pathology, National Relevance Hospital “S.G. Moscati”, Avellino, Italy; 3 Department of Biochemistry and Medical Biotechnology–CEINGE, “Federico II” University, Naples, Italy; 4 Molecular Oncology Unit, IRCCS, “Centro di Riferimento Oncologico della Basilicata”, Rionero in Vulture, Pz, Italy; 5 Department of Cellular and Molecular Biology and Pathology, “Federico II” University, Naples, Italy; INSERM- CNRS- Univ. Méditerranée, France

## Abstract

The thymus is the primary organ able to support T cell ontogeny, abrogated in FOXN1^−/−^ human athymia. Although evidence indicates that in animal models T lymphocytes may differentiate at extrathymic sites, whether this process is really thymus-independent has still to be clarified. In an athymic FOXN1^−/−^ fetus, in which we previously described a total blockage of CD4^+^ and partial blockage of CD8^+^ cell development, we investigated whether intestine could play a role as extrathymic site of T-lymphopoiesis in humans. We document the presence of few extrathymically developed T lymphocytes and the presence in the intestine of CD3^+^ and CD8^+^, but not of CD4^+^ cells, a few of them exhibiting a CD45RA^+^ naïve phenotype. The expression of CD3εεpTα, RAG1 and RAG2 transcripts in the intestine and TCR gene rearrangement was also documented, thus indicating that in humans the partial T cell ontogeny occurring at extrathymic sites is a thymus- and FOXN1-independent process.

## Introduction

The thymus supports a proper T cell ontogeny due to the presence of specialized epithelial cells, resulting in the export of naïve CD45RA^+^ CD62L^+^ T cells that follows the recruitment of progenitors from bone marrow [1].

Evidence indicates that T cells may also differentiate at extrathymic sites, as intestine and liver [2]–[6], where T cell populations may arise from preexisting precursor cells [7], [8], even though it still remains to be demonstrated if the process is fully thymus-independent. In favor of a thymic independent differentiation process there is the evidence that a few T cells can be detected into the periphery in nude mice [9]–[11]. The T cell pool developed outside the thymus exhibits a peculiar phenotype [2] although not univocal in the different species. In fact, in mice, extrathymic T cells often exhibit the CD8αα homodimer, while in rats they may be CD8αβ [12]. In human fetal intestine, T cells are characterized by a higher proportion of TCRγδ^+^ and CD8αα^+^ cells [13]. In addition, CD4 and CD8 double negative T cells (CD3^+^CD4^–^CD8^–^) isolated from the intestine are generally considered of extrathymic origin [13]. In the epithelium of the small intestine, lymphocytes may also express CD7 and CD2 in the absence of CD3 (CD2^+^CD3^–^CD7^+^). In humans, the expression of RAG in the gut indicates that at this site a gene rearrangement process may take place, suggesting an active lymphopoiesis [14].

FOXN1 is a developmentally regulated transcription factor, selectively expressed in epithelial cells of the skin and thymus, where it plays a necessary role for T lymphopoiesis [15]–[17] by inducing a proper epithelial cell differentiation and endothelial cell/thymic mesenchyme communication network [18]. FOXN1 mutations lead to athymia [19], [20] and result, in humans, in a SCID phenotype, referred as the human equivalent of the mice Nude/SCID syndrome [21]–[24]. During early prenatal life in humans, homozygous FOXN1 mutation leads to a complete blockage of the CD4^+^ T cell maturation, while a few CD8α^+^TCRγδ^+^ cells, not expressing CD3ε molecule and not able to respond to a mitogenic stimulation, are found [25], thus suggesting an extrathymic site of lymphopoiesis for these cells.

Here we studied the role of the intestine and liver as extrathymic sites of thymus-independent and FOXN1-independent T lymphopoiesis in a FOXN1^−/−^ athymic human fetus. We found the presence of a few T cells with a peculiar phenotype, indicative of the thymus-independent lymphopoiesis.

## Results and Discussion

### Detection of extrathymically derived T lymphocytes in the cord blood of FOXN1^−/−^ fetus

The fetus analyzed in the present study was identified during a genetic counseling offered to heterozygous couples at risk for Nude/SCID disease, originated in the same geographic area where the first patients were identified [26]. The specific defect (R255X mutation in the FOXN1 gene) was searched on chorionic villi by direct sequencing.

**Figure 1:**
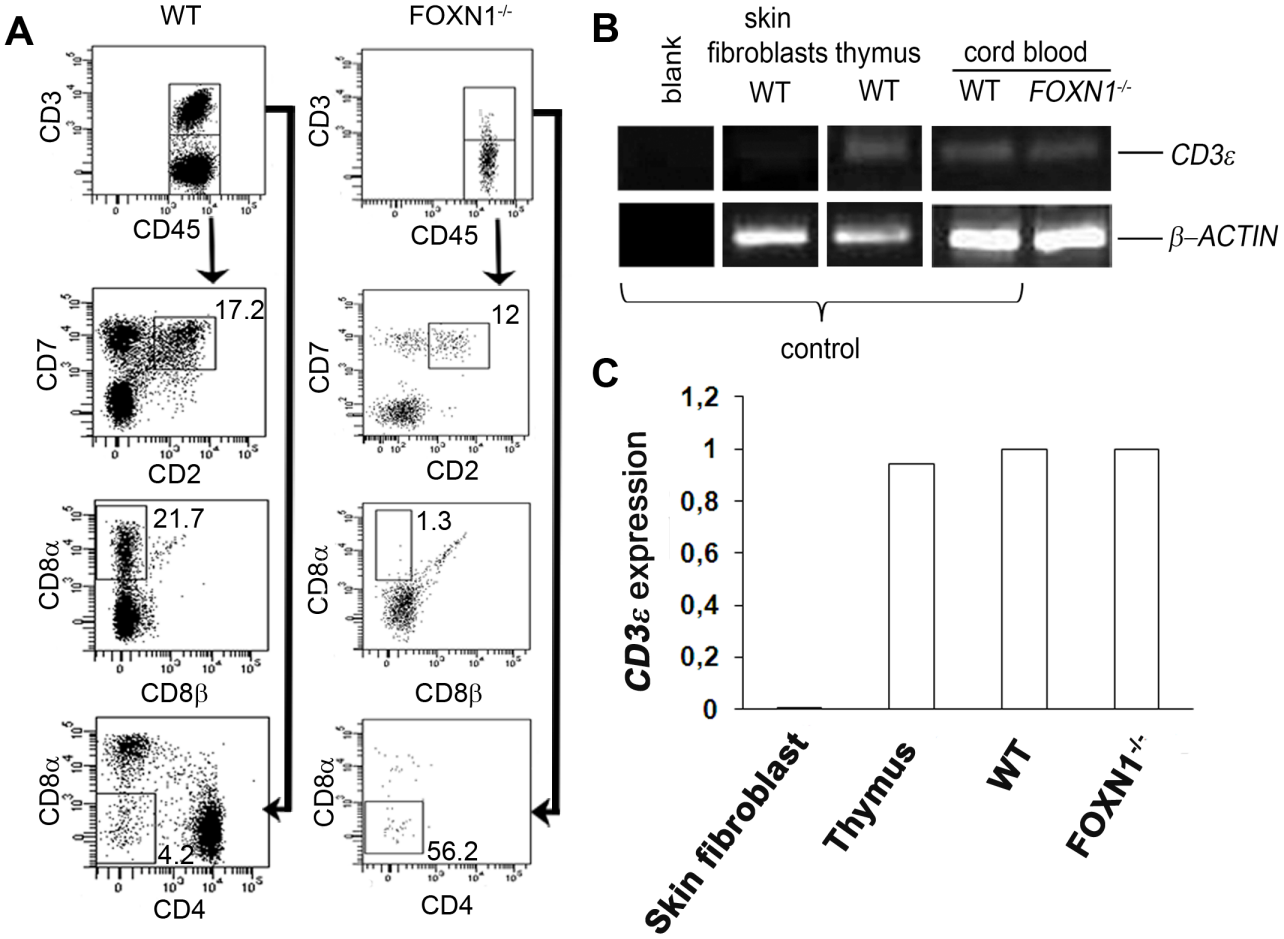
Detection of extrathymically derived T lymphocytes in the cord blood of FOXN1^−/−^ human fetus. (A) Flow cytometry analysis of CBMCs from WT (left dot plots) or FOXN1^−/−^ (right dot plots) fetuses (16 weeks of gestation). CD7 and CD2 together with the CD8α and CD8β expression patterns for the gated CD45^+^CD3^−^ cells are shown. CD8α and CD4 expression is shown for the gated CD45^+^CD3^+^ cells. Numbers indicate the frequency of the cells within the gate. Experiment was repeated two times. Data were obtained by gating first on viable cells and later on CD45^+^ cells. (B) RT-PCR analysis of CD3ε expression in CBMCs. The expression of CD3ε transcript in human skin fibroblasts (negative control), human thymus (positive control), CBMCs from WT or FOXN1^−/−^ fetuses is shown. Blanck, no cDNA. β-actin was used as loading control. Representative results from three independent experiments are shown. (C) Quantitative real-time PCR showing the expression of mRNAs encoding CD3ε (relative to β-actin) in skin fibroblasts (negative control), thymus (positive control) and CBMCs from WT or FOXN1^−/−^ fetuses (16 weeks of gestation). Representative results from two independent experiments are shown.

In the absence of the thymus, few lymphocytes in CB co-express CD7^+^CD2^+^ (12% of CD3^−^ gated lymphocytes) in the FOXN1^−/−^ fetus, as compared to the control (17.2%) (Figure 1A). This population also comprises NK cells.

Extrathymic derived intraepithelial lymphocytes (IELs) are difficult to be univocally characterized, in that in mice they preferentially bear TCRγδ and express the CD8αα [11], [27], [28], while in rats they express the CD8αβ heterodimer [12]. We previously described that in the FOXN1^−/−^ CBMCs, most of the CD8^+^ cells were CD3^−^ [25], thus we looked at the CD8αα^+^ cells on CD3^−^ gated lymphocytes. These cells were 1.3% in the FOXN1^−/−^ CBMCs and much more represented in the control (21.7%) (Figure 1A). Our data are in favor of a thymic dependence of such cells. In nude mice, a number of TCR^+^CD8αα^+^ T IELs, lower than what found in euthymic mice, has also been reported [11].

The absence of CD4 molecule, would argue against the possibility that the CD3^−^CD8α^+^ cells were dendritic cells (DC) [29]. Moreover, since the CD3^−^CD8α^+^ cells were analyzed setting the gate on lymphoid cells, this would rule out the possibility that they were DCs of myeloid origin. In addition, the CD3^−^CD8α^+^ cells are unlikely to be NK cells, in that they should express the CD8 with a dim intensity instead of a CD8 with a bright intensity, as in T cells, similarly to what found in Nude/SCID fetus.

In the FOXN1^−/−^ fetus, most of the rare CD3^+^ cells were CD4 and CD8 double negative (56.2% of CD3^+^ gated lymphocytes) as compared to the control (4.2% of CD3^+^ gated lymphocytes) (Figure 1A). A novel population of T cells with a similar phenotype, CD3^+^B220^low^CD4^−^CD8^−^, has also been identified in a *nu/nu* mouse, suggesting an extrathymic origin [30].

We previously documented in the FOXN1^−/−^ human fetus a considerable number of CD3ε^−^CD8α^+^TCRγδ^+^ cells, which also comprises cells with the CD8αβ heterodimer [25]. IELs may express only a partial CD3 complex bearing rare message of the ε chain although the T cell commitment is established by the presence of pTα transcript [31], [32]. Thus, we evaluated the median fluorescence intensity (MFI) of CD3 signal, which was much lower in the FOXN1^−/−^ fetus than in the control (213 versus 1275 MFI, respectively), in keeping with the already reported dim signal in the same FOXN1^−/−^ fetus [25]. The presence in the FOXN1^−/−^ fetus of CD3^+^ cells, was however confirmed by the presence of the CD3ε transcript (Figure 1B). It should be noted that mRNA expression is almost equivalent in both FOXN1^−/−^ cells and wild type (Figure 1C). Thus, we cannot exclude that CD3^−^ cells are really CD3ε^ low^ cells.

### Extrathymic sites of B-, NK- and T-lymphopoiesis in FOXN1^−/−^ SCID human fetus

**Figure 2:**
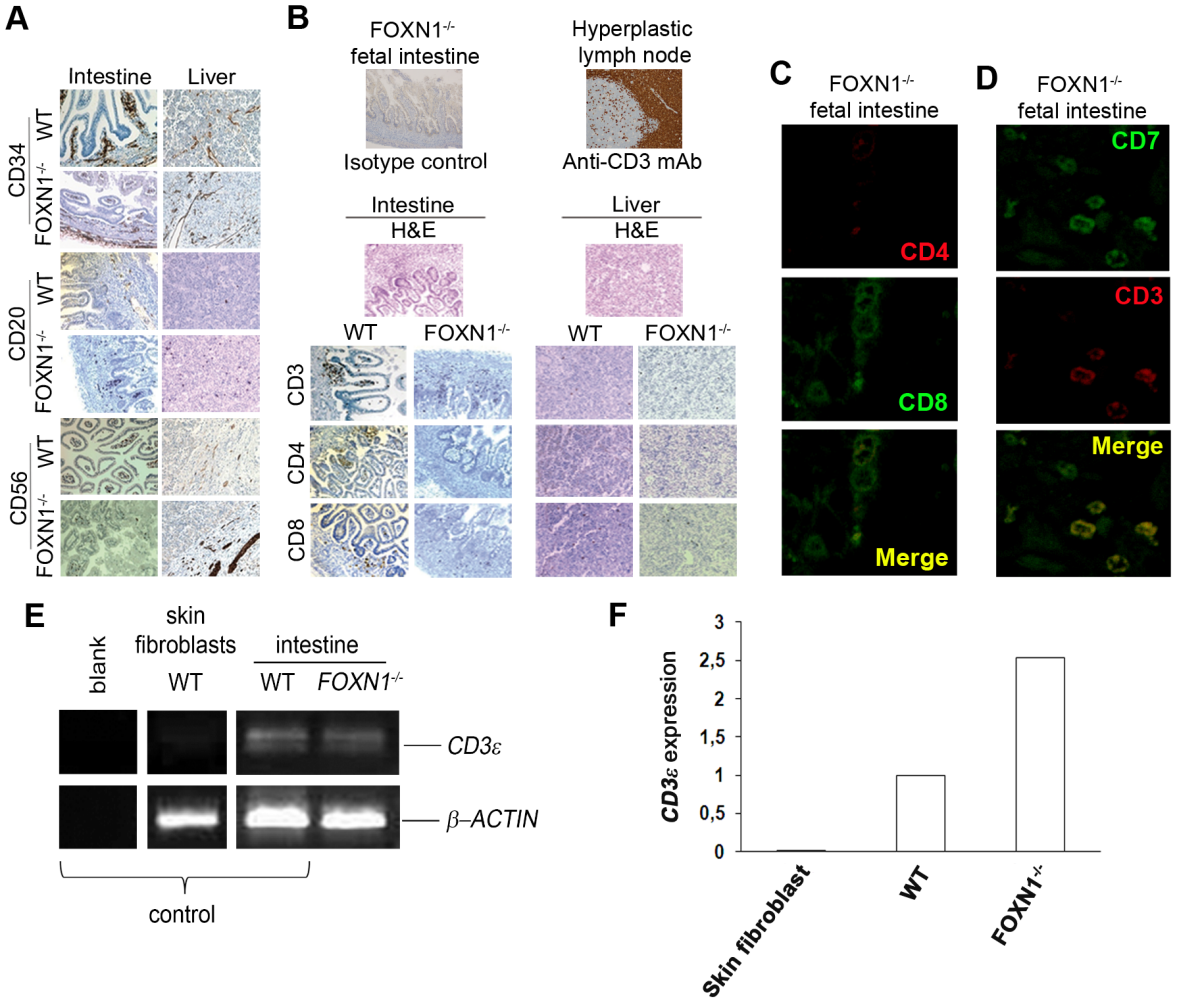
Identification of lymphocytes at extrathymic sites of differentiation in a Nude/SCID human fetus. (A, B) Immunohistochemical detection of lymphocytes at extrathymic sites of differentiation in FOXN1^−/−^ human fetus (16 weeks of gestation). (A) Stem cells, B cells and NK cells were detected by immunohistochemical stain for CD34 (brown), CD20 (brown) and CD56 (brown) in intestinal and liver sections obtained from a FOXN1^−/−^ human fetus (16 weeks of gestation) or an aged-matched control fetus. In CD34 and CD20 stained intestine sections from WT and FOXN1^−/−^ fetuses original magnification was 100x. (B) As negative control, intestinal sections from FOXN1^−/−^ fetus were counterstained with hematoxylin and with the isotype control (primary antibody omitted) by DAB. As positive control, hyperplastic lymph node sections were stained for CD3 (brown) by DAB. Intestinal or liver sections of FOXN1^−/−^ human fetus were counterstained with hematoxylin and eosin (H&E). Immunohistochemical analysis of intestinal or liver sections of a FOXN1^−/−^ human fetus and an aged-matched control fetus using anti-CD3 staining to mark T cells, anti-CD4 staining to mark T helper cells and anti-CD8 staining to mark cytotoxic T cells. DAB, 200x. Representative results from two independent experiments with two distinct samples are shown. (C, D) Confocal microscopy of FOXN1^−/−^ intestinal sections. (C) Labeling with anti-human CD4 (red) and anti-human CD8 (green). (D) Labeling with anti-human CD7 (green) and anti-human CD3 (red). Representative results from three independent experiments are shown. (E) RT-PCR analysis of CD3ε intestinal expression. CD3ε transcript expression in human skin fibroblasts (negative control), intestinal lymphocytes from WT or FOXN1^−/−^ fetuses is shown. Blanck, no cDNA. β-actin was used as loading control. Representative results from three independent experiments are shown. (F) Quantitative real-time PCR showing the expression of mRNAs encoding CD3ε (relative to β-actin) in skin fibroblasts (negative control), thymus (positive control) and intestinal tissue of control and FOXN1^−/−^ fetuses (16 weeks of gestation). Representative results from two independent experiments are shown.

Since in humans, intestine and liver are considered the main organs for extrathymic lymphopoiesis [2], we characterized the lymphocytes in these tissues. As expected on the T^low^B^+^NK^+^ phenotype of the human Nude/SCID [21], CD34^+^ cells and B cells (CD20) were normal in tissue sections (Figure 2A). The CD56 marker for NK cells revealed the presence of few and spread positive cells in the intestine sections but not in the liver (Figure 2A). Eventually, these findings confirm that, at 16 weeks of gestation, the development of mature B and NK cells is a thymus-independent process. Moreover, also the morphology of intestine and liver sections, evaluated through H&E staining, was normal (Figure 2B).

Within FOXN1^−/−^ intestine tissue, CD3^+^ cells were spread in the mucosa with a trend to aggregate in the crypts while in the control they formed clear aggregates (Figure 2B). In the liver of both FOXN1^−/−^ and control, CD3^+^ cells were present but spread (Figure 2B). Accordingly to what found in CB, CD4^+^ cells were absent in either intestine and liver of the FOXN1^−/−^ fetus, differently from the control (Figure 2B). A few CD8^+^ cells were detected in the FOXN1^−/−^ intestine similarly to the control (Figure 2B). Quantification in 5 random fields of the positive cells, stained as in Figure 2B, confirmed the absence of CD4^+^ cells and the presence of few CD8^+^ cells in both tissues of the FOXN1^−/−^ fetus (19.6±3 in FOXN1^−/−^ intestine versus 27.4±2 in WT intestine, *p* ≤ 0.05; 9.8±2 in FOXN1^−/−^ liver versus 14.6±4 in WT liver). No double positive (CD4^+^CD8^+^) thymocytes were found by confocal microscopy in the FOXN1^−/−^ intestine (Figure 2C). When CD7^+^ cells were also stained for CD3, a few CD3^−^CD7^+^ cells were detected in the intestine, even though the majority of them co-expressed both molecules (Figure 2D). In the FOXN1^−/−^ fetus we demonstrated the presence of the CD3ε transcript through RT-PCR amplification of intestinal mRNA (Figure 2E). The quantitative PCR analysis revealed that the amount of this molecule in the intestine of FOXN1^−/−^ fetus was even higher than in the control (Figure 2F). Taken together these data suggest that a local production of T lymphocytes takes place in the intestine and liver in a thymus- and FOXN1-independent manner, even though we cannot completely exclude an early contribution of a thymus primordium to the production of T cells.

### Cells with naive phenotype can develop in the FOXN1^−/−^ human athymic fetus

**Figure 3:**
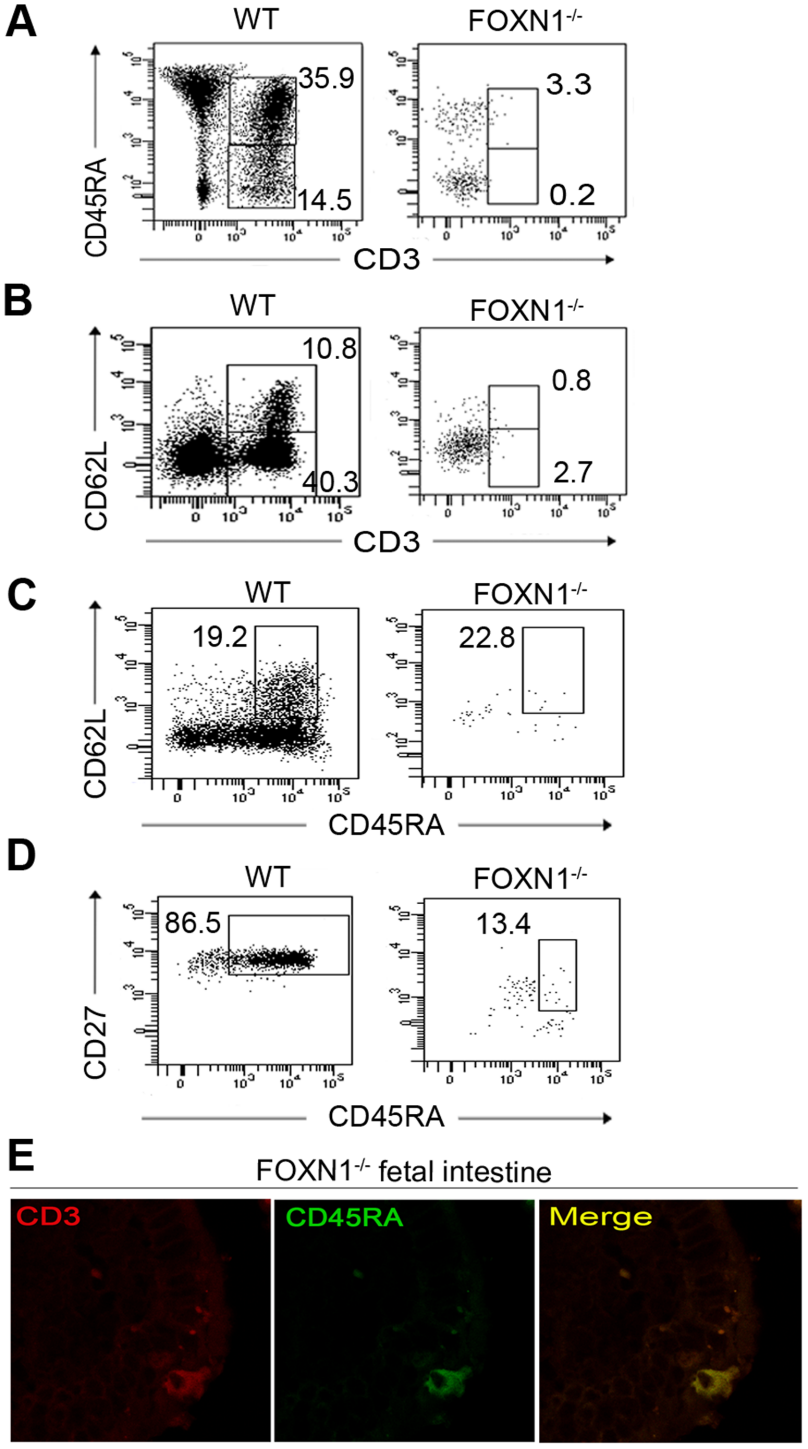
Lymphocytes with naive phenotype in cord blood and intestine. Flow cytometry of CBMCs from normal and FOXN1^−/−^ fetuses matched for gestational age (16 weeks of gestation). Dot plots show the expression pattern of the naïve cell markers. (A) Frequencies of CBMCs expressing both CD45RA and CD3 markers. (B) Frequencies of CBMCs expressing both CD62L and CD3 markers. (C) Frequencies of CBMCs coexpressing CD45RA and CD62L markers. (D) Frequencies of CBMCs coexpressing CD45RA and CD27 markers. Experiment in (A), (B), (C) and (D) was repeated two times. Data were obtained by gating first on viable cells and later on CD45^+^ cells (A and B) or finally also on CD3^+^ (C and D). (E) Confocal microscopy of fetal intestinal sections labeled with anti-human CD3 (red) and anti-human CD45RA (green). Representative results from three independent experiments with two samples are shown.

The CD45RA molecule and the L-Selectin CD62L are considered markers of Recent Thymic Emigrants (RTE), thus being the hallmark of naïve lymphocytes. In FOXN1^−/−^ CBMCs, 3.3% of CD45^+^ gated cells co-expressed CD3, with a dim intensity, and the CD45RA, differently from the control, in whom this population was 35.9%, almost all expressing CD3 with bright intensity (Figure 3A). On CD45^+^ gated cells, only a negligible number co-expressed the CD3 and CD62L markers (0.8%), as compared to the 10.8% of the control (Figure 3B). In FOXN1^−/−^ CBMCs, 22.8% of CD3^+^ cells co-expressed both CD62L and CD45RA, similarly to the control (Figure 3C). The analysis of CD27 associated with CD45RA, as a further marker of a naïve cell phenotype [33], [34], revealed the presence of CD27^+^CD45RA^+^ cells (13.4% of CD3^+^ gated cells) in FOXN1^−/−^ CBMCs (Figure 3D). The immunofluorescence co-staining of CD45RA and CD3 molecules revealed in the intestine of the FOXN1^−/−^ fetus the presence of cells co-expressing both molecules (Figure 3E). This finding indicates that intestinal T lymphocytes also exhibit a naive phenotype.

### Identification of intestinal de novo lymphopoiesis in the FOXN1^−/−^ athymic fetus

**Figure 4:**
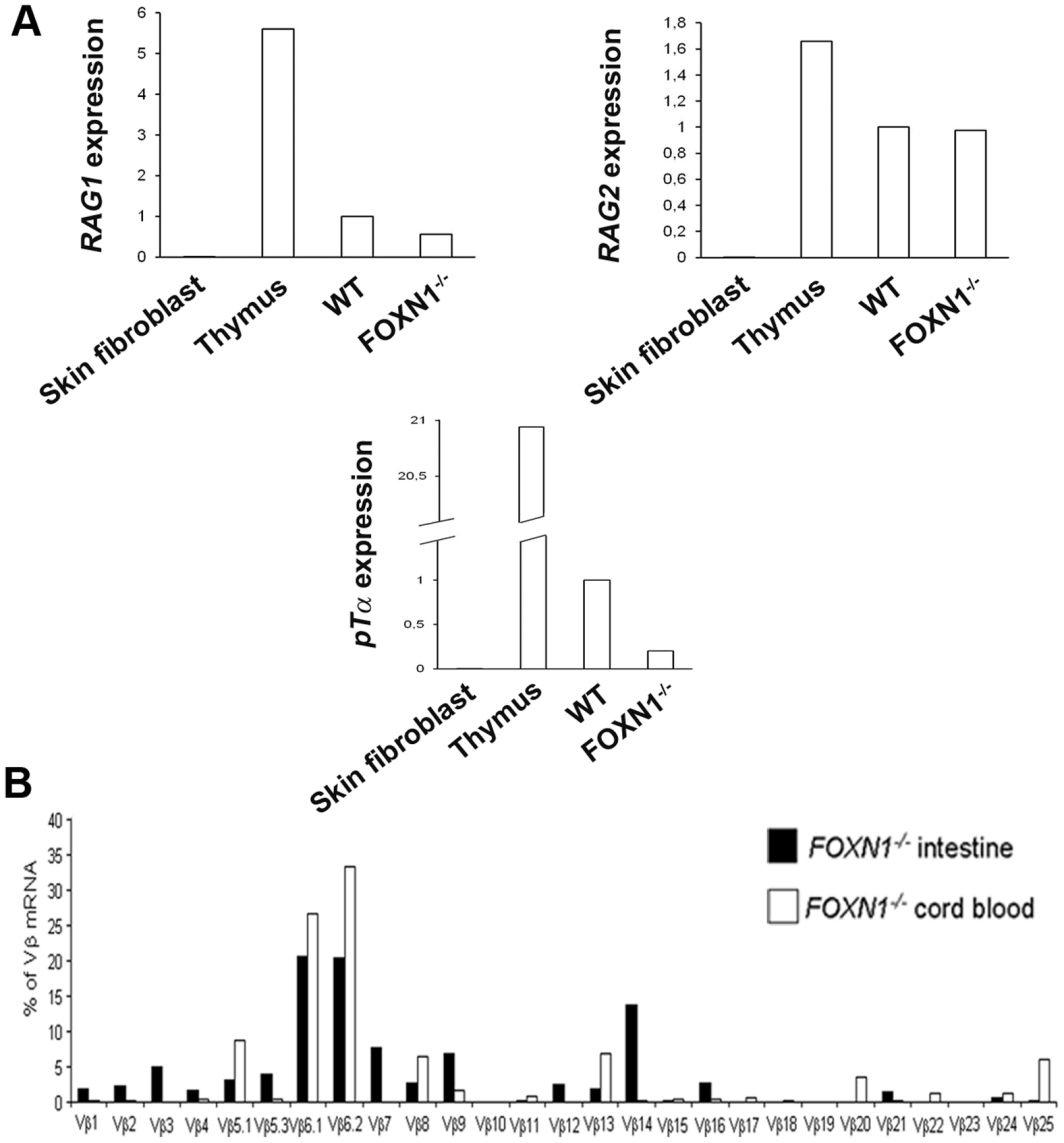
Local production of T cells in the FOXN1^−/−^ human intestine. (A) Quantitative real-time PCR showing the expression of mRNAs encoding RAG1, RAG2 and pTα (relative to β-actin) in skin fibroblasts (negative control), thymus (positive control) and intestinal tissue of control and FOXN1^−/−^ fetuses (16 weeks of gestation). (B) Comparison of TCR Vβ-region usage between intestinal lymphocytes (black bars) and CBMCs (white bars) from the FOXN1^−/−^ fetus. Experiments were repeated three times in (A) and two times in (B).

TCR gene rearrangement occurs at the T cell precursor stage and results in a functional antigen receptor. The process requires RAG1 and RAG2 recombination activity, which results in pTα production. The fate of pTα-expressing progenitors was found to include all αβ and most γδ T cells but to exclude B, NK, and thymic dendritic cells [32]. The expression of the surrogate TCR chain pTα is upregulated during the DN3 stage of the T lymphocyte development, along with the expression of the RAG genes. pTα expression is also found in pro-T cells at extrathymic sites of the T cell development in Nude mice [35]. Also in IELs RAG1 and pTα mRNAs are expressed, thus indicating an ongoing TCR gene rearrangement locally in the intestine [14], [36]. In the FOXN1^−/−^ fetus, the relative expression of RAG1 and RAG2 mRNA was 47.5 and 68.4% of the control, respectively (Figure 4A), whereas pTα even though to a lesser extent, is detectable, accounting for 20.0% of the control (Figure 4A), thus suggesting that in the absence of the thymus the rearrangement occurs in the FOXN1^−/−^ intestine, but the process is only limited to few T cells. These results, along with the CD3ε expression, suggest the presence of a de novo intestinal production of T cells. While RAG1 and RAG2 enzymes are highly expressed also in pro-B and pre-BII cells, the expression of the pTα is in favor of a thymus-independent T-lymphopoiesis.

In this study, we found that the TCR repertoire of FOXN1^−/−^ intestinal lymphocytes paralleled the CBMCs spectratype, which was consistently impaired [25]. A statistically significant quantitative correlation was found in the contribution of all Vβ families to the TCR repertoire (*r*  =  0.78; *p* < 0.001), but of Vβ14, which represented the only family, with an intestinal expression higher than 10% of the total mRNA, not being expressed at all in CBMCs (Figure 4B). The lower expression of the pTα in the FOXN1^−/−^ intestine, as compared to the control, along with the altered TCR spectratyping, suggest that only a partial T cell ontogeny occurs in the intestine, limited to very few families and resulting in a limited repertoire, generated in a thymus-independent fashion.

**Figure 5:**
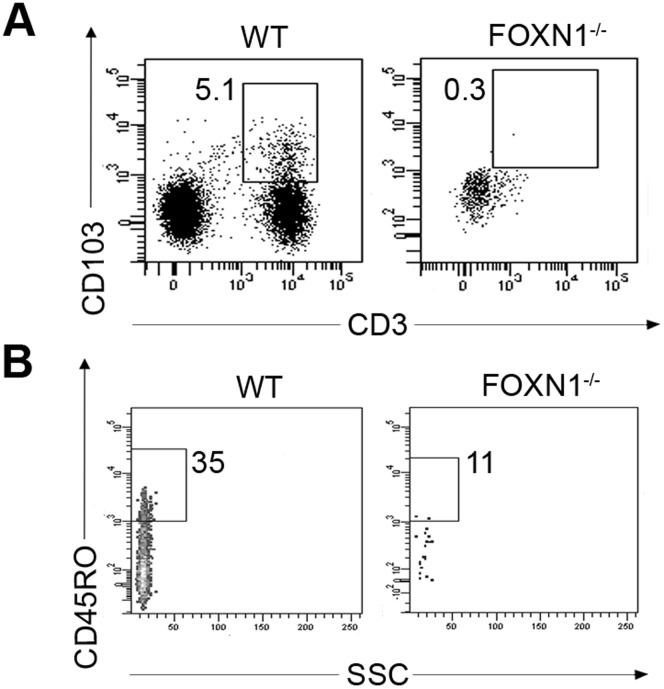
Expression on T lymphocytes from FOXN1^−/−^ and control fetuses of the integrin CD103 and the CD45RO activation marker. (A, B) Flow cytometry of CBMCs from normal and FOXN1^−/−^ fetuses matched for gestational age (16 weeks of gestations). Dot plots show the frequency of CD103^+^CD3^+^ on CD45^+^ gated CBMCs (A) and CD45RO^+^ on CD3^+^ gated CBMCs (B).

T cells localized in the epithelium of skin, gut, lung and allograft tissues are characterized by the expression of the αEβ7 integrin CD103, which is involved in directing previously stimulated lymphocytes, above originated, to epithelial cells [37]. No CD3^+^CD103^+^ cells were detected in both FOXN1^−/−^ fetus CB (0.3% of CD45^+^ gated cells) (Figure 5A), and at the immunohistochemical evaluation of FOXN1^−/−^ intestine tissue (not shown), suggesting that intestinal T cells in the FOXN1^−/−^ fetus were locally produced. Moreover, in the FOXN1^−/−^ CBMCs, most of T cells didn’t express the CD45RO activation marker (Figure 5B), indicating that they had not previously encountered any antigen.

In conclusion, although there is still the possibility that a thymus anlage contributes to produce the few T cells observed in the FOXN1^−/−^ patients, our results support the hypothesis that T cells do mature at extrathymic sites with an alternative lymphopoietic process, involving the same molecules implicated in intrathymic development, as pTα and RAGs. This process in humans is thymus- and FOXN1-independent. In summary, we document that a few T lymphocytes with a peculiar phenotype may develop in a thymus- and FOXN1-independent manner. We also report on the presence of intestinal CD3^+^ and CD8^+^, but not CD4^+^ cells, a few of them showing a naïve phenotype. The expression of CD3εε pTα, RAG1 and RAG2 transcripts in the intestine and TCR gene rearrangement, although abnormal, indicates that in humans a partial T cell ontogeny occurs at extrathymic sites in the Nude/SCID phenotype in a FOXN1-independent manner.

## Materials and Methods

### Fetus samples

Cord blood (CB) from the FOXN1^−/−^ fetus was obtained by cordocentesis at 16 weeks of gestation. Experiments using CB or fetal tissue samples were approved by the Institutional Ethical Committee for Biomedical Activities “Carlo Romano” at the “Federico II” University of Naples. Age-matched CB cells from the CEINGE bank were used as control. Fetus parents provided written informed consent.

### Cell-surface staining and flow cytometry

In flow cytometry, FITC-, phycoerythrin (PE)-, allophyocyanin-cyanine 7 (APC-Cy7)-, peridin chlorophyll protein (PerCP)- or PE-Cy7-coupled Abs were used on CB toward CD45 (2D1), CD7 (M-T701), CD2 (RPA-2.10), CD3 (UCHT1), CD8α (SK-1), CD8β (2ST8.5H7), CD4 (L200), CD62L (SK11), CD45RA (HI100), CD27 (L128), CD45RO (UCHL-1), CD103 (Ber-ACT8) from BD Pharmingen, San Diego, CA or Beckman Coulter, Brea, CA. FACSCanto II flow cytometer and FACSDiva software (BD Bioscience, San Jose, CA) were used. For each sample, negative controls were stained with irrelevant Abs conjugated with the same fluorochrome [38]. The “fluorescence-minus-one” (FMO) controls have also been used to define precisely the cells that have fluorescence above background levels. Briefly, the samples have been stained with all of the reagents except one [38].

### Histology

Intestine and liver tissue samples from a 16 weeks FOXN1^−/−^ fetus or control were embedded in OCT compound and snapfrozen in liquid nitrogen or paraffin-embedded. The blocks were cut into serial 5-μm sections and mounted onto microscope slides for H&E staining and immunohistochemistry analysis. Immunodetections were performed by means of a Ventana automat (Ventana Medical Systems, Illkirch, France).

### Immunohistochemistry

Tissue sections staining was performed on Benchmark XT platform (Ventana Medical Systems) with pre-diluted CD34, CD20, CD56, CD3, CD4 (Ventana-Confirm), CD8 (Cell Marque), 1:40 CD103 (Beckman Coulter, Brea, CA), 1:50 CD45RA (Dako, Denmark) and 1:25 CD62L Abs (Abcam, Cambridge, UK). Heat antigen retrieval was performed in buffer (CC1, Ventana) following the manufacturer instructions. The slides were incubated with primary Abs at 37° for 32 min (CD34, CD20, CD56, CD3, CD4 and CD8) or for 60 min (CD45RA, CD103, CD62L). Primary Ab was omitted for negative control. Nuclei were counterstained with hematoxylin. The reaction was detected by the ultraView Universal DAB Detection Kit, which utilizes a cocktail of enzyme labeled secondary Abs that locates the bound primary Ab. The complex is then visualized with hydrogen peroxide substrate and 3, 3′-diaminobenzidine tetrahydrochloride (DAB) chromogen, which produces a dark brown precipitate readily detected by light microscopy. Images were acquired by a microscope (DM 2500; Leica, Germany) at magnification 200 x or 100 x.

### Confocal microscopy

Tissue samples were blocked with normal goat serum before staining and then treated with 1∶50 of PerCP-labeled CD3 (BD Pharmingen, San Diego, CA) and 1∶100 of FITC-labeled CD45RA Abs (BD Pharmingen, San Diego, CA) or 1∶50 of PE-labeled CD4 (Beckman Coulter, Brea, CA) and 1∶50 FITC-labeled CD8 (Beckman Coulter, Brea, CA) or 1∶50 APC-labeled CD3 (Beckman Coulter, Brea, CA) and FITC-labeled CD7 (Beckman Coulter, Brea, CA). Images were acquired by a confocal microscope (LSM 510, Zeiss, Germany).

### RNA and RT-PCR

Total RNA was isolated from normal human skin fibroblasts, normal human thymus, CB mononuclear cells (CBMCs) or intestinal frozen tissue using TRIzol reagent (Invitrogen, Carlsbad, CA) and the Phase-lock gel columns (Eppendorf) by standard procedures. RNA was reverse transcribed by SuperScript III reverse transcription (Invitrogen, Carlsbad, CA). RT-PCR was performed using Taq polymerase (Roche, Germany). The following primers were used to amplify CD3ε: (forward) 5′-GATGCAGTCGGGCACTCACT-3′ and (reverse) 5′-TTGGGGGCAAGATGGTAATG-3′); or β*-*actin: (forward) 5′-GACAGGATGCAGAAGGAGAT-3′ and (reverse) 5′-TTGCTGATCCACATCTGCTG-3′. To avoid amplification of genomic DNA, the reverse primer for CD3ε was located on the 3-4 exons junction.

### Evaluation of TCR β-chain variable region (Vβ) spectratyping

TCR CDR3β sequencing of total mRNA isolated from intestine of the FOXN1^−/−^ or control fetuses was performed after TCR β-chain amplification with a common reverse primer (CB3 primer) and 27 different forward primers (TCR Vβ gene family primers). PCR products were run on a CEQ 8000 automatic capillary sequencer (Beckman Coulter, Brea, CA) and fractionated on the size of the CDR3 region. Results were analyzed using CEQ 8000 software (Beckman Coulter, Brea, CA), which also gives the percentage contribution of a single family to the total TCR repertoire.

### Quantitative real-time PCR

Real-time PCR was performed using the SYBR green detection reagent and analyzed with the Light Cycler480 system (Roche, Germany). Genes were normalized to β*-*actin as housekeeping gene and the relative messenger RNA expression data were analyzed using the 2^−ΔΔCt^ method [39]. The following primers were used to amplify β*-*actin: (forward) 5′-GACAGGATGCAGAAGGAGAT-3′ and (reverse) 5′-TTGCTGATCCACATCTGCTG-3′; or CD3ε: (forward) 5′-GATGCAGTCGGGCACTCACT-3′ and (reverse) 5′-TTGGGGGCAAGATGGTAATG-3′; or RAG1: (forward) 5′-CATCAAGCCAACCTTCGACAT-3′ and (reverse) 5′-CAGGACCATGGACTGGATATCTC-3′; or RAG2: (forward) 5′-CCTGAAGCCAGATATGGTC-3′ and (reverse) 5′-GTGCAATTCACAGCTGGGCT-3′; or pTα: (forward) 5′-CATCCTGGGAGCCTTTGGT-3′ and (reverse) 5′-CCGGTGTCCCCCTGAGAG-3′. The pTα reverse primer was located on the 3-4 exons junction to avoid DNA contamination.

### Statistical analysis

GraphPad Prism software was used for data analysis. The *t*-student test was used to analyze the statistical significance of differences. The minimum acceptable level of significance was *p* ≤ 0.05.
